# Supplementary material for: Fuse to defuse: a self-limiting ribonuclease-ring nuclease fusion for type III CRISPR defence
Source: Nucleic Acids Res. 2020 Apr 29;48(11):6149–56. doi: 10.1093/nar/gkaa298 (PMC7293037; doi:10.1093/nar/gkaa298)
Supplement: gkaa298_Supplemental_File [file gkaa298_supplemental_file.pdf]

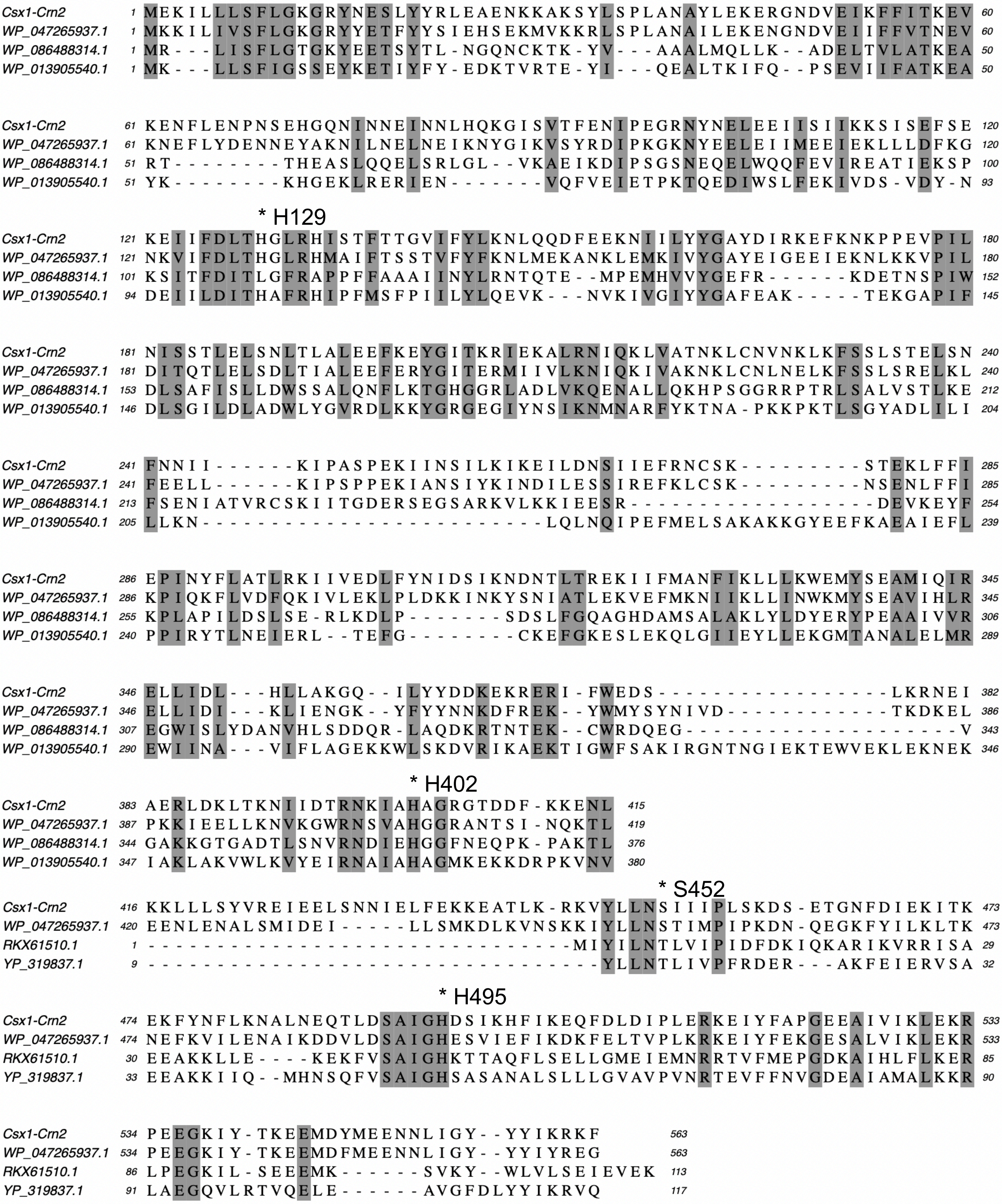

Supplementary figure 1. Multiple sequence alignment of Csx1-Crn2 with selected homologues. *M. piezophila* Csx1-Crn2 is aligned with the sole bifunctional homologue in NCBI, WP_047265937.1 from a related strain; with Csx1 homologues from *Thioflexothrix psekupsii* (WP_086488314.1) and *Pyrococcus yayanosii* (WP_013905540.1); and with Crn2 homologues from *Thermodesulfobacteria* bacterium (RKX61510) and Acidianus two-tailed virus (YP_319837.1). Residues mutated in this study are indicated.

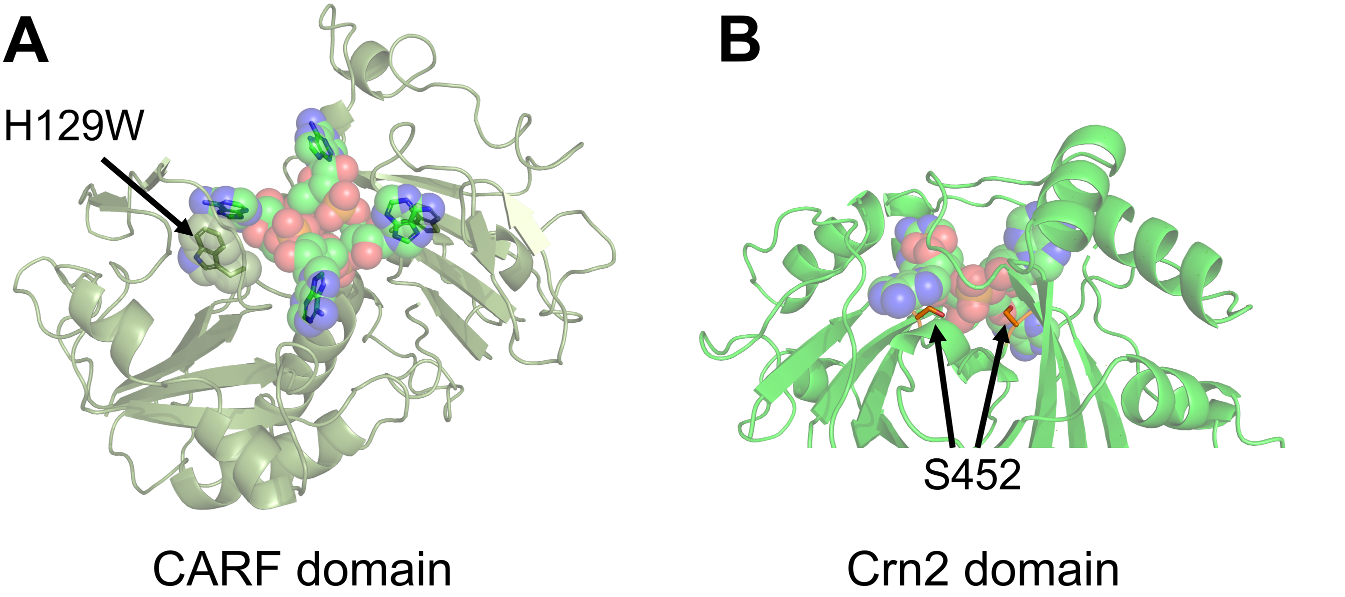

Supplementary figure 2. Mutations designed to abolish cA4 binding in the CARF and Crn2 domains. A The CARF domain of the Csx1-Crn2 structure model is shown with cA_4 bound, based on the structure of Csx1 from *S. islandicus* (PDB 6R7B). Mutation of H129 to a tryptophan is predicted to result in a steric clash with the adenine base of cA4. **B** The Crn2 domain of the Csx1-Crn2 structure model is shown with cA_4 bound, based on the structure of AcrIII-1 from SIRV1 (PDB 6SCF). S452 is conserved in the Crn2 family and mutation to a bulkier tyrosine residue is predicted to block cA_4 binding. Colouring is consistent with that used in Figure 1.
